# The characteristics of general practitioners and geriatricians who take overall responsibility for the care of older patients with multimorbidity

**DOI:** 10.1002/jgf2.727

**Published:** 2024-09-18

**Authors:** Takuma Kimura, Shinji Matsumura, Masayoshi Hashimoto, Ken Shinmura

**Affiliations:** ^1^ Department of R&D Innovation for Home Care Medicine Tokyo Medical and Dental University School of Medicine Tokyo Japan; ^2^ Department of General Medicine Tokyo Medical and Dental University School of Medicine Tokyo Japan; ^3^ Matsumura Clinic Tokyo Japan; ^4^ Division of Clinical Epidemiology National Hospital Organization Tokyo Medical Center Tokyo Japan; ^5^ Department of General Internal Medicine Hyogo Medical University, School of Medicine Hyogo Japan

**Keywords:** family medicine specialists, geriatric specialists, multimorbidity, older patients, primary care‐certified physicians

## Abstract

**Background:**

Older patients with multimorbidity often seek care from multiple health care providers and visit several medical institutions. Having a primary care provider who takes overall responsibility for their care may be beneficial. We conducted a survey to identify the characteristics of general practitioners and geriatricians who frequently assume such responsibility for older patients with multimorbidity.

**Methods:**

In June 2022, we distributed by mail an anonymous questionnaire to 3300 family medicine specialists, primary care‐certified physicians, and geriatric specialists in Japan. We used a four‐point Likert‐type scale to score items related to conditions and patient backgrounds that pose challenges in treatment, key clinical factors, and important clinical strategies. Modified Poisson regression was used to identify factors associated with frequently assuming overall responsibility for medical care.

**Results:**

Data from 746 physicians were included in the analysis. Factors associated with frequently assuming overall responsibility for medical care of older patients with multimorbidity included providing inpatient ward care (prevalence ratio [PR] 1.237, 95% CI 1.124–1.362), providing home medical care (PR 1.357, 95% CI 1.225–1.504), frequently treating patients over 90 years old (PR 2.043, 95% CI 1.258–3.318), and the overall score for clinical strategies (PR 1.021, 95% CI 1.010–1.033).

**Conclusions:**

General practitioners and geriatricians who frequently assume overall responsibility for the care of older patients with multimorbidity tend to engage significantly in ward and home medical care, often treat patients above 90 years, and employ numerous clinical strategies.

## Abstract 2

Factors associated with taking responsibility for medical care for older patients with multimorbidity included working at a clinic, providing care on a ward, providing home medical care, frequently working with patient over 90 years old, and overall score for clinical strategies.

## 1 INTRODUCTION

In recent years, the prevalence of multimorbidity among older adults has risen, posing challenges in identifying a primary condition.^1^ In Japan, multimorbidity is associated with increased mortality, heightened polypharmacy, and patterns that diminish the health‐related quality of life of these individuals.^2^, ^3^, ^4^, ^5^, ^6^

Older patients with multimorbidity often visit multiple medical institutions and consult numerous physicians.^7^, ^8^ This situation often leads to the absence of a dedicated primary care provider who views the patient holistically and coordinates their overall medical care. This absence can be detrimental to patients.^7^, ^8^ A survey conducted among physicians at clinics in Japan found that only 20% were aware of all the medical facilities their patients visited,^2^ suggesting a scarcity of primary care providers who oversee the overall care of older patients with multimorbidity. The number of older patients with multimorbidity is expected to rise, highlighting the need to increase the number of physicians who take comprehensive responsibility for this population. Strategies to improve care include individualizing treatment plans, adopting integrated approaches, enhancing decision support, and designating a lead physician to oversee and coordinate care.^9^, ^10^, ^11^, ^12^

In Europe and the United States, generalists are often appropriate as the primary care provider with overall responsibility for managing the care of older patients with multimorbidity, for several reasons.^3^, ^4^, ^5^, ^6^ First, not all comorbidities in older patients are linked to a single specific condition, making a specialist generally less suitable as the primary care provider.^8^ Second, older patients with multiple conditions frequently visit multiple medical facilities and receive fragmented care from multiple physicians, underscoring the need for a single physician to assume overall responsibility for their medical care.^9^, ^10^, ^13^ Michael Balint, in the 1950s, emphasized the importance of one physician managing care when multiple providers are involved.^8^, ^14^ He highlighted the “collusion of anonymity” in which both general practitioners and specialists avoid taking responsibility for patients, each assuming that someone else will do so.^14^ In recent years, the rise in the number of older adults with multimorbidity and the corresponding increase in health care practice fragmentation have increased the importance of appointing someone to ensure a comprehensive approach to this population. This includes taking responsibility for their immediate and future health care needs, coordinating care, and advocating on their behalf. This role also involves maintaining the continuity of information and overall care management.^10^

In both Europe and the United States, general practitioners and geriatricians—physicians most likely to be engaged in the management of medical care for older patients with multimorbidity—function as generalists and primary care providers.^15^, ^16^ A survey in the UK showed that many older adults with multiple chronic conditions often consult their GPs, who feel a strong sense of responsibility for their care.^9^, ^17^ This sense of responsibility is likely enhanced by the institutionalized family doctor system in the UK, a concept also gaining attention in Japan as discussions about institutionalizing the function of primary care providers unfold.^18^ It would be beneficial for Japan to explore policy support aimed at fostering responsibility for the care of older adults with multimorbidity.^19^ However, Japan faces challenges in this regard, as there is often no single primary care provider responsible for the overall care of these patients, whether in community settings or during hospital admissions.^7^ This issue is exacerbated by a shortage of general practitioners and geriatric specialists who would typically fill these roles. As of April 2022, the Japanese Primary Care Association (JPCA), a professional organization for general practitioners, reported having only 1091 family medicine specialists and 5435 primary care‐certified physicians. Similarly, the Japan Geriatrics Society, which focuses on geriatric medicine, has only 1650 geriatric specialists. Furthermore, it is often unclear who should be designated as the primary care provider, a decision that requires agreement between patients and physicians within the Japanese health care system.^19^ Internists are considered to be part of the group of physicians responsible for treating patients with multimorbidity. However, Japanese internists often specialize in specific organs or fields of medicine. Therefore, we targeted general practitioners and geriatricians in our study.

It is therefore necessary to support and increase the number of general practitioners and geriatricians in Japan who will serve as primary care providers for older patients with multimorbidity. However, the attributes of these professionals currently fulfilling this role are not well understood. Gaining insight into these characteristics could inform the development of strategies and educational programs aimed at increasing the number of physicians prepared to act as primary care providers for this patient group.

The increase in the number of patients with multimorbidity is closely linked to the increase in the number of older people.^6^ We believe that the issue of having responsibility for treating older patients with multimorbidity in Japan, the world's most‐aged population, will be of great concern in other countries with aging populations, even if their health care systems differ from that of Japan.

Accordingly, we conducted a survey in Japan to explore the differences in medical backgrounds and approaches among general practitioners and geriatricians involved in the management of older patients with multimorbidity. Specifically, we examined the differences between those who often assume overall responsibility for the patient's care as “primary care providers” and those who do not.

## 2 METHODS

### 2.1 Participants

Between June and July 2022, we carried out an anonymous survey using a questionnaire distributed via postal mail to 3300 participants. These participants included 1650 primary care specialists, randomly selected from a pool of 1091 family medicine specialists and 5435 primary care‐certified physicians affiliated with the JPCA (total membership of 11,506). We also included all geriatric specialists from the Japan Geriatric Society (JGS; total membership of 6541), amounting to 1650.

### 2.2 Questionnaire

The questionnaire consisted of two parts: approaches to treating older patients with multimorbidity and respondent backgrounds.^20^ The questions on treatment approaches were informed by prior research and discussions among the research team.^9^, ^10^, ^11^, ^12^, ^15^, ^16^, ^21^, ^22^, ^23^ The model is based on the idea that when treating older patients with multimorbidity, physicians encounter conditions or patient histories that they find more challenging. The importance of clinical backgrounds and strategies in the treatment process varies among physicians, influencing the complexity of care and their readiness to assume the role of the primary care provider.

#### 2.2.1 Approaches to treating older patients with multimorbidity

The questionnaire evaluated four aspects of treating older patients with multimorbidity. The first aspect involves patient conditions that complicate the treatment of multimorbidity (conditions), probed by the question, “When treating older patients (65 years and older) with multimorbidity, how difficult do you feel it is to treat the following conditions?” (43 items, Appendix 1). The second aspect covers patient backgrounds that complicate treatment (backgrounds), addressed with the question, “When treating older patients (65 years and older) with multimorbidity, how difficult do you feel it is to treat patients with the following backgrounds?” (14 items, Appendix 2). The third aspect pertains to clinical factors that are important in managing multimorbidity (clinical factors), addressed by the question, “When treating older patients (65 years and older) with multimorbidity, how important are the following clinical elements?” (32 items, Appendix 3). The fourth aspect involves clinical strategies that are important when treating multimorbidity (clinical strategies), posed by the question, “When treating older patients (65 years and older) with multimorbidity, how important are the following clinical strategies?” (19 items, Appendix 4). Participants were asked to mark each response on a Likert‐type scale ranging from 1 = “not at all” to 4 = “very much.”

Additional questions included, “Do you find it more difficult to treat older patients with multimorbidity than other patients?” (difficulty of treatment), and the main outcome, “Do you have overall responsibility for the treatment of older patients with multimorbidity?” (responsibility for treatment). Responses were recorded on a four‐point Likert‐type scale from “not at all” to “very much.”

#### 2.2.2 Background of the respondents

The questionnaire collected demographic and professional data from respondents, including their gender, age, and length of experience as physicians (years and months). It also inquired about their workplace, offering options such as “non‐bedded clinic,” “bedded clinic,” “hospital with fewer than 200 beds,” “hospital with more than 200 beds,” “university hospital,” or “long‐term care facilities.” In addition, they were asked about the clinical settings of their work, allowing for multiple answers such as “outpatient clinics,” “home medical care,” “long‐term care facilities,” and “wards”. The respondents were also asked about the population size of the municipality in which they practiced and the frequency with which they treated age groups 65–74, 75–89, and 90 years and older, with response options ranging from “never,” “not often,” “sometimes,” and “often.”^24^

### 2.3 Analysis

#### 2.3.1 Data analysis

##### Approaches to treating older patients with multimorbidity

To evaluate various aspects of patient management, scores from four categories—conditions, background, clinical factors, and clinical strategies—were aggregated. The conditions category used a 172‐point scale, with higher scores indicating an increased number of patient conditions perceived as challenging to treat (Overall Score for Difficult Conditions). For background, this was on a 56‐point scale, with higher scores indicating more patient backgrounds that are considered difficult to treat (Overall Score for Difficult Backgrounds). The score for clinical factors was based on a 128‐point scale, with higher scores indicating a greater emphasis on a broad range of clinical factors (Overall Score for Important Clinical Factors). Lastly, the clinical strategies score was based on a 76‐point scale, with higher scores indicating a broader consideration of clinical strategies (Overall Score for Important Clinical Strategies).

For the “degree of clinical difficulty,” respondents who answered either “often” or “sometimes” were classified into the “difficult” group, and those who answered “not often” or “not at all” were categorized into the “not difficult” group.

The main outcome focused on the physician's perspective on assuming “medical responsibility” frequently, an attribute desirable in a primary care provider. Therefore, those who answered “often” were classified into the “frequently responsible” group, and those who answered “sometimes,” “not often,” or “not at all” were classified into the “other” group for further analysis.

##### Background of the respondents

The population size of the practice location was classified based on a population of 100,000. The population of 100,000 is used in Japan as a unit of measurement when describing the number of doctors and serves as the threshold between small‐ and medium‐sized cities under Japan's Local Autonomy Law. The “under 100,000” group included those whose municipality had a population of <50,000 or between 50,000 and 100,000. The “over 100,000” group included those in municipalities with populations ranging from 100,000 to 300,000, 300,000 to 500,000, or >500,000.

The frequency with which physicians treated patients aged 65–74, 75–89, and over 90 years old was grouped into low frequency for participants who answered with “never” or “not often,” and high frequency for those who answered “sometimes” or “often.”

#### 2.3.2 Statistical analysis

##### Background of the respondents

We used the Chi‐square test, Fisher's exact test, for categorical variables and the *t‐*test for continuous variables to evaluate differences in the overall responsibility among the “frequently responsible” and the “other” groups based on gender, age, years of experience as a physician, facility, clinical setting, population size, frequency of practice by age groups.

##### Approach to treating older people with multimorbidity

Differences between the “frequently responsible” and “other” groups were assessed by their overall score for difficult conditions, difficult backgrounds, important clinical factors, and important clinical management, as well as by clinical difficulty (“difficult” or “not difficult”), using the chi‐square test for categorical variables and *t*‐test for continuous variables. We generated the hypothesis that a lower overall score for difficult conditions/difficult backgrounds and higher overall score for important clinical factors/clinical strategies are associated with physicians being “frequently responsible.”

##### Factors related to frequently taking overall responsibility for the care of older people with multimorbidity

Using the status of frequently assuming overall responsibility for the care of older individuals with multimorbidity (=1) as the outcome, according to recommendations for statistical analysis of binary outcomes, modified Poisson regression was performed to calculate prevalence ratios (PRs) and 95% confidence intervals (CIs).^25^ Our analysis model, agreed upon by the research team, included the following explanatory variables: years of experience as a physician, clinical setting (outpatient, ward, home medical care, long‐term care facility), population size, frequency of practice involving patients older than 90 years, approaches to treating older patients with multimorbidity (overall score for difficult conditions, difficult backgrounds, important clinical factors, and important clinical strategies) and clinical difficulty. We employed forced entry for the variables, conducted two‐tailed tests, set significance levels at less than 5%, and used the statistical package IBM SPSS Statistics 29.0.1.

## 3 RESULTS

**FIGURE 1:**
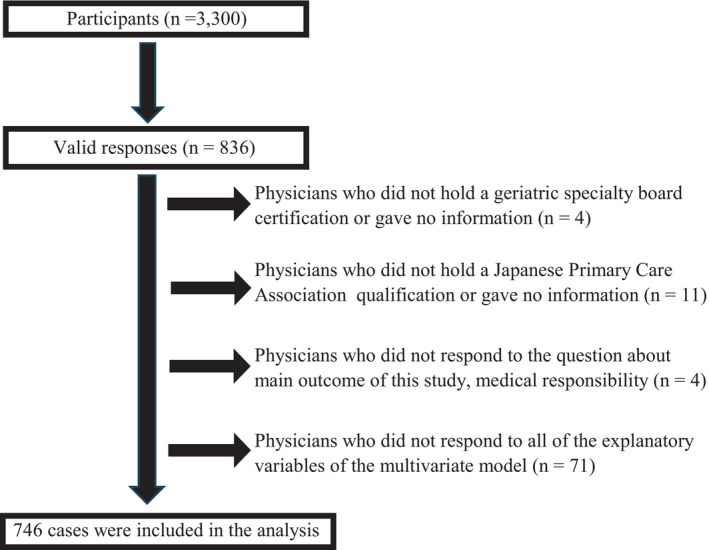
Population flow diagram of the study participants.

There were 836 valid responses (25.3% response rate). This included 11 respondents who identified themselves as primary care specialists but were neither a family medicine specialist nor a primary care‐certified physician, or who did not respond to this question. In addition, four respondents who claimed to be geriatric specialists but either lacked a geriatric specialist qualification or did not respond to that question were also excluded. After removing these 15 respondents and an additional four participants who did not respond to the study's main outcome regarding medical responsibility, 746 individuals remained for the final analysis. These participants had complete data for all explanatory variables of the multivariate model described above (Figure 1).

The mean age of participants was 53.53 ± 12.11 years, and the average years of experience as a physician was 32.14 ± 12.03 years. Of the respondents, 606 (81.7%) were male and 136 (18.3%) were female. Regarding the type of facility, 224 (32.3%) worked in clinics and 469 (67.7%) in hospitals.

In terms of taking responsibility for the overall care of older patients with multimorbidity, 519 (69.6%) of the doctors reported that they “often” did so, 200 (26.8%) “sometimes,” 24 (3.2%) “not often,” and 3 (0.4%) “not at all.” We classified 519 (69.6%) respondents into the “frequently responsible” group and 227 (30.4%) in the “other” group.

Concerning the difficulty in treating older patients with multimorbidity, 340 (45.6%) respondents “often” found it difficult, 342 (45.8%) “sometimes,” 64 (8.6%) “not often,” and none (0%) “never.” This categorization placed 682 (91.4%) respondents in the “difficult” group and 64 (8.6%) in the “not difficult” group.

### 3.1 Backgrounds of respondents (Table 1)

**TABLE 1:** Respondents' backgrounds (*n* = 746).

	Level of responsibility for care of older people with multimorbidity				
	Frequently responsible (*n* = 519, 69.6%)		Other (*n* = 227, 30.4%)		*p* Value
	*N* or mean	% or SD	*N* or mean	% or SD	
Gender					0.248
Male	415	80.6%	191	84.1%	
Female	100	19.4%	36	15.9%	
Age	52.5	11.85	56.0	12.35	<0.001
Experience as a physician (years)	26.8	11.60	30.3	12.04	<0.001
Type of facility					<0.001
Non‐bedded clinic	151	30.4%	50	23.0%	
Bedded clinic	21	4.2%	2	0.9%	
Hospital with <200 beds	127	25.6%	46	21.2%	
Hospital with >200 beds	132	26.6%	75	34.6%	
University hospital	58	11.7%	31	14.3%	
Long‐term care facilities	6	1.2%	7	3.2%	
Clinical setting					
Outpatient					0.639
Provided	487	93.8%	215	94.7%	
Not	32	6.2%	12	5.3%	
Home medical care					<0.001
Provided	248	47.8%	38	16.7%	
Not	271	52.2%	189	83.3%	
Long‐term care facility					<0.001
Provided	170	32.8%	38	16.7%	
Not	349	67.2%	189	83.3%	
Ward					0.001
Provided	299	57.6%	101	44.5%	
Not	220	42.4%	126	55.5%	
Population size of the municipality					0.025
Over 100,000	365	70.5%	178	78.4%	
Under 100,000	153	29.5%	49	21.6%	
Frequency of treating patients aged 65 to 75 years					1.000
High	514	99.4%	225	99.6%	
Low	3	0.6%	1	0.4%	
Frequency of treating patients aged 75 to 90 years					0.028
High	519	100.0%	223	98.7%	
Low	0	0.0%	3	1.3%	
Frequency of treating patients aged 90 or older					<0.001
High	508	97.9%	199	87.7%	
Low	11	2.1%	28	12.3%	

Abbreviation: SD, standard deviation.

Overall, there was no significant difference in gender between the “frequently responsible” and “other” groups. The “frequently responsible” group was statistically younger and had fewer years of experience as physicians compared with the “other” group.

The “frequently responsible” group was also statistically more likely than the “other” group to provide home medical care, treatment in long‐term care facilities, and inpatient ward care. In addition, two significant differences in respondent characteristics included a higher likelihood of being in the “other” group for those in municipalities with populations under 100,000, and a greater tendency for the “frequently responsible” group to treat patients over 90 years old.

### 3.2 Approach to treating older patients with multimorbidity (Table 2)

**TABLE 2:** Approach to treating older people with multimorbidity (*n* = 746).

	Level of responsibility for care of older people with multimorbidity				
	Frequently responsible (*n* = 519, 69.6%)		Other (*n* = 227, 30.4%)		*p* Value
	*N* or mean	% or SD	*N* or mean	% or SD	
Conditions score	110.75	17.93	113.04	16.62	0.100
Patient background score	43.76	6.96	44.07	6.35	0.569
Important Clinical factors score	103.44	11.43	100.82	10.63	0.003
Clinical strategies score	62.68	6.52	59.65	5.40	<0.001
Degree of clinical difficulty					0.881
Not difficult	44	8.5%	20	8.8%	
Difficult	475	91.5%	207	91.2%	

There were no statistically significant differences in overall scores for managing difficult conditions and patient backgrounds between the two groups. However, overall scores for important clinical factors and clinical strategies were statistically higher in the “frequently responsible” group compared with the “other” group. There was no significant difference in the perception of clinical difficulty between the groups.

### 3.3 Factors associated with assuming overall responsibility for the treatment of older patients with multimorbidity (Table 3)

**TABLE 3:** Factors related to frequently taking overall responsibility for the care of older people with multimorbidity (*n* = 746).

	Prevalence ratio	95% confidence interval		*p* Value
		Lower	Higher	
Experience as a physician (years)	0.998	0.994	1.002	0.300
Clinical setting				
Outpatient				
Provided	0.969	0.811	1.159	0.730
Not	Reference	–	–	
Ward				
Provided	1.237	1.124	1.362	<0.001
Not	Reference	–	–	
Home medical care				
Provided	1.357	1.225	1.504	<0.001
Not	Reference	–	–	
Long‐term care facility				
Provided	1.071	0.978	1.173	0.141
Not	Reference	–	–	
Population size of the municipality				
Under 100,000	0.976	0.889	1.071	0.603
Over 100,000	Reference	–	–	
Frequency of treating patients aged 90 years or older				
High	2.043	1.258	3.318	0.004
Low	Reference	–	–	
Conditions score	0.999	0.996	1.003	0.752
Patient background score	0.995	0.986	1.004	0.260
Important Clinical factors score	1.000	0.994	1.006	0.948
Clinical strategies score	1.021	1.010	1.033	<0.001
Degree of clinical difficulty				
Not difficult	1.021	0.858	1.215	0.818
Difficult	Reference	–	–	

Modified Poisson regression revealed factors associated with frequently assuming overall responsibility for the care of older patients with multimorbidity: the PR for providing ward‐based care was 1.237 (95% confidence interval [CI]: 1.124–1.362), and for home medical care, it was 1.357 (95% CI: 1.225–1.504). The PR for providing care at high frequency to those aged 90 and older was 2.043 (95% CI: 1.258–3.318) for the frequently responsible group. In addition, a one‐point increase in the overall clinical strategies score was associated with a 2.1% increase in the likelihood of frequently assuming responsibility (PR 1.021, 95% CI: 1.010–1.033).

To determine the presence of multicollinearity, we checked the Kendall correlation coefficients among all independent variables entered into the analytical model and found no correlations with *τ* > 0.70 (maximum of 0.597).

## 4 DISCUSSION

To the best of our knowledge, this is the first study to explore the roles of primary care and geriatric specialists in Japan, aiming to identify factors associated with frequently taking overall responsibility for the medical care of older patients with multimorbidity. These results could inform strategies to promote responsibility of care for patients with multimorbidity.

The provision of both inpatient and home‐based medical care was associated with frequently taking overall responsibility for the medical care of older patients with multimorbidity. In the context of inpatient ward care, studies have found that generalists, such as general practitioners and geriatricians, play crucial roles as primary care providers. This includes care coordination and interprofessional collaboration during hospitalization.^18^ Patients receiving home medical care typically present with more complex needs compared with those in hospital settings because of the number of comorbidities and level of cognitive impairment.^26^, ^27^ Home medical care is indicated for individuals who have difficulty accessing hospital services, making it imperative that their care is managed holistically by a dedicated primary care provider.

Taking responsibility was associated with a high frequency of caring for those aged 90 years and older. It is important to consider both functional assessments and chronological age for treatment indications, yet general practitioners and geriatricians often perceive a need to assume greater overall responsibility for patients with multimorbidity in this age group.

Our findings revealed that frequently assuming overall responsibility did not correlate with the overall score for conditions, patient backgrounds, or clinical factors, which contradicted our hypothesis. However, as we hypothesized, a higher overall score for clinical strategies was associated with increased responsibility‐taking. This suggests that overall care management for older adults with multimorbidity involves the patients, their families, specialists, primary care provider, and various professionals. The use of a wide range of clinical strategies was significantly correlated with assuming overall responsibility for care, suggesting that strengthening the education and training of individual physicians may be an effective way to increase their willingness to assume care responsibility for this patient group. Additional research is necessary to determine whether improvements in clinical strategies, such as those identified in this study, will encourage more physicians to take responsibility for the care of these patients.

This study had several limitations. The first concerns its generalizability. The study included physicians who were randomly selected from either JPCA family medicine specialists or primary care‐certified physicians, as well as all geriatric specialists across Japan. This cohort was considered central to the management of older patients with multimorbidity in Japan, implying a degree of representativeness. However, with a response rate of only 25.3%, interpreting the results requires caution. The respondents might represent only those who are highly interested in the management of older patients with multimorbidity, potentially skewing the insights toward those without such interest. Second, in this study, the responsibility in clinical practice was measured based on physicians' self‐assessment. Thus, there may be a discrepancy between self‐assessed responsibility and the evaluation by patients or other health care professionals. In addition, we used original questionnaires and scales for explanatory variables, which have not been validated. Third, the study employed the Japanese term *sekinin* to describe a physician managing the overall medical care of older patients with multimorbidity. Given the ambiguity of the term “primary care provider” in the Japanese medical system, the survey specifically asked if physicians assumed *sekinin*, a term that encompasses both “responsibility” and “accountability” in English. This dual interpretation might have introduced some ambiguity regarding the question's intent.^28^, ^29^ However, we believe that both the attributes of responsibility and accountability are required for a physician acting as a primary care provider for this patient group, justifying the use of *sekinin* in this context.

The findings of this study have important implications for future medical education and policy development. They support the development of educational programs focused on clinical strategies and settings, as well as the establishment of policies that promote responsibility in ward or home medical care practices. Although this was a cross‐sectional study and causality cannot be established, the experience of providing care on the ward and home medical care, as well as the experience of treating very elderly patients, may promote responsible care for patients with multimorbidity. Therefore, increasing such clinical experiences in specialty training programs of family medicine or geriatrics might lead to an increase in the number of responsible primary care providers.

## 5 CONCLUSIONS

Among family medicine specialists, primary care‐certified physicians, and geriatric specialists in Japan, 69.6% frequently assume overall responsibility for the care of older patients with multimorbidity. These physicians typically engage more intensively in ward care and home care, care for patients over 90 years of age, and use a variety of clinical strategies.

## CONFLICT OF INTEREST STATEMENT

Takuma Kimura has received research funding and grants (C.U.C. Inc.). The other authors have stated explicitly that there are no conflicts of interest in connection with this article.

## ETHICS STATEMENT

This study was approved by the Ethical Review Committee of the Maruki Memorial Medical and Social Welfare Center, the first author's former institution (No. 37). The cover page of the questionnaire provided an outline of the survey, its purpose, privacy protection, and contact information.

## PATIENT CONSENT STATEMENT

Consent was inferred upon the return of the completed questionnaire.

## APPENDIX 1 DISEASES THAT CAUSE DIFFICULTIES WHEN TREATING OLDER PATIENTS WITH MULTIMORBIDITY

### 1.1

1. Congestive heart failure.
2. Hypertension.
3. Atrial fibrillation.
4. Ischemic heart disease (with history of acute myocardial infarction or hospitalization for catheterization and treatment).
5. Peripheral arterial disease (including intermittent claudication, postbypass, gangrene or untreated thoraco‐abdominal aortic aneurysm of >5 cm).
6. Diabetes mellitus (severe: presence of any of the three major complications or a history of hospitalization for diabetic ketoacidosis or diabetic coma).
7. Diabetes mellitus (mild: absence of the three major complications, excluding diet management alone).
8. Dyslipidemia.
9. Gout/hyperuricemia.
10. Thyroid disease.
11. Chronic lung disease (dyspnea with light exertion).
12. Chronic kidney disease (CKD grade 5, GFR <15, Cre ≥3 mg/dL, on dialysis, post‐renal transplant, or uremia).
13. Cerebrovascular disease (mild sequelae, history of cerebrovascular disease without sequelae, history of transient ischemic attack).
14. Hemiplegia (including paraplegia, even if not caused by cerebrovascular disorder).
15. Intractable neurological disease.
16. Peptic ulcer.
17. Inflammatory bowel disease.
18. Constipation.
19. Collagen disease.
20. Liver disease (mild liver cirrhosis without portal hypertension, chronic hepatitis).
21. Moderate‐to‐severe hepatic dysfunction (liver cirrhosis with portal hypertension).
22. Solid tumor (no apparent metastasis in the past 5 years).
23. Cancer metastasis/metastatic solid cancer.
24. Lymphoma (including lymphosarcoma, macroglobulinemia, myeloma).
25. Leukemia or true erythrocytosis.
26. Depression.
27. Dementia.
28. Sleep disorders.
29. Benign prostatic hyperplasia.
30. Vertigo.
31. Hearing loss.
32. Low back pain.
33. Epilepsy.
34. Spinal canal stenosis.
35. Osteoporosis.
36. Osteoarthritis.
37. Visual impairment.
38. Glaucoma.
39. Cataract.
40. Dental problems.
41. Bedsores.
42. Eczema/dermatitis.
43. AIDS.

## APPENDIX 2 PATIENT BACKGROUNDS THAT CAUSE DIFFICULTIES WHEN TREATING OLDER PATIENTS WITH MULTIMORBIDITY

### 2.1

1. Severe comorbidities.
2. Multiple social problems.
3. Many psychiatric/psychological problems.
4. Difficulty in setting the goals and outcomes of medical treatment.
5. Difficulty communicating with patients.
6. Difficulty communicating with family members.
7. No key person is available.
8. Patient lives alone.
9. Difficulty in identifying the department or medical institution where the patient is receiving concurrent treatment.
10. Difficulty collecting clinical information from outpatient visits to other clinics or hospitals.
11. Difficulty collecting clinical information during admissions or discharges from other hospitals.
12. Unable to follow general practice guidelines.
13. Difficulty collaborating with a specialist in specific organs or fields.
14. Differences of opinion on goal setting with specialists in specific organs or fields.

## APPENDIX 3 CLINICAL FACTORS THAT ARE IMPORTANT WHEN TREATING OLDER PATIENTS WITH MULTIMORBIDITY

### 3.1

1. Comorbidities in the primary disease.
2. Hearing loss.
3. Visual impairment.
4. Use of a wheelchair in everyday life.
5. Being bedridden in everyday life.
6. Cognitive impairment.
7. Depression.
8. Low nutrition.
9. Psychiatric complications.
10. Urinary incontinence.
11. History of falls.
12. Certified as requiring long‐term care insurance.
13. Poor adherence to medications.
14. Polypharmacy.
15. Burdened by waiting time for outpatient departments.
16. Burdened by visits to the doctor and outpatient departments.
17. Decrease in quality of life (QOL) from exercise therapy.
18. Decrease in QOL from dietary therapy.
19. Decrease in QOL from medications.
20. Burden of treatment.
21. Limited support for daily living.
22. Decreased ability to care for the patient at home.
23. Repeated emergency room visits.
24. Repeated hospitalizations and discharges.
25. Financial problems.
26. Living alone.
27. Absence of a physician who coordinates and decides overall medical policy.
28. Number of medical institutions attended.
29. Number of coexisting diseases.
30. Age.
31. Estimated life expectancy.
32. Frailty.

## APPENDIX 4 CLINICAL STRATEGIES THAT ARE IMPORTANT WHEN TREATING OLDER PATIENTS WITH MULTIMORBIDITY

### 4.1

1. Refer to practice guidelines with older adults in mind.
2. Review evidence on important outcomes.
3. Review indications for drug therapy for primary prevention.
4. Review indications for drug therapy for secondary prevention.
5. Re‐evaluate medications.
6. Reassess treatment strategy.
7. Evaluate patient treatment burden.
8. Listen to the patient's wishes and values.
9. Listen to the opinions of the family.
10. Ask the opinions of other professionals.
11. Ask for opinions from people the patient trusts.
12. Present options to the patient for treatment goals.
13. Present treatment priorities to the patient.
14. Consolidate the number of physicians who will provide treatment.
15. Identify a physician who will determine the overall treatment plan.
16. Examine the intervals between visits to organ/field specialist.
17. Clarify the role of the organ/field specialist.
18. Use of long‐term care insurance services.
19. Multidisciplinary intervention.
